# Investigation of Surface Integrity Induced by Ultra-Precision Grinding and Scratching of Glassy Carbon

**DOI:** 10.3390/mi14122240

**Published:** 2023-12-14

**Authors:** Kirk Jahnel, Robert Michels, Dennis Patrick Wilhelm, Tim Grunwald, Thomas Bergs

**Affiliations:** 1 Department of Fine Machining and Optics, Fraunhofer Institute for Production Technology IPT, 52074 Aachen, Germany; robert.michels@ipt.fraunhofer.de (R.M.); dennis.patrick.wilhelm@ipt.fraunhofer.de (D.P.W.); tim.grunwald@ipt.fraunhofer.de (T.G.); t.bergs@wzl.rwth-aachen.de (T.B.); 2 Chair for Manufacturing Technology, Tool Machine Laboratory (WZL), RWTH Aachen University, 52074 Aachen, Germany

**Keywords:** ultra-precision grinding, nano-scratch, surface integrity, ductile grinding, brittle material, glassy carbon

## Abstract

Glassy carbon provides material characteristics that make it a promising candidate for use as a mould material in precision glass moulding. However, to effectively utilize glassy carbon, a thorough investigation into the machining of high-precision optical surfaces is necessary, which has not been thoroughly investigated. This research analyses the process of material removal and its resulting surface integrity through the use of nano-scratching and ultra-precision grinding. The nano-scratching process begins with ductile plastic deformation, then progresses with funnel-shaped breakouts in the contact zone, and finally concludes with brittle conchoidal breakouts when the cutting depth is increased. The influence of process factors and tool-related parameters resulting from grinding has discernible impacts on the ultimate surface roughness and topography. Enhancing the cutting speed during cross-axis kinematic grinding results in improved surface roughness. Increasing the size of diamond grains and feed rates leads to an increase in surface roughness. An achievable surface roughness of Ra < 5 nm together with ductile-regime grinding behaviour meet optical standards, which makes ultra-precision grinding a suitable process for optical surface generation.

## 1. Introduction

In contemporary times, a substantial quantity of sensors is integrated into widely used commodities such as smartphones and automobiles. A significant portion of these sensors rely on MEMS due to their compact dimensions and efficient energy usage, which enable a wide range of sensory functions. The integration of borosilicate glass optical components, such as protective coverings and beam guides, with MEMS, is facilitating the development of compact optoelectronic applications [1]. These systems are made possible because of the thermal expansion properties of borosilicate glass types, which are specifically tailored to be compatible with silicon. As a result, the risk of stress formation during the joining process can be mitigated. Modern applications necessitate the use of intricate glass components with advanced optical properties. Precision glass moulding is a well-established technological process utilized for the effective production of optics. This process involves the shaping of a glass preform using moulds that possess a high degree of precision [2,3]. However, the moulding temperature of borosilicate glass (>700 °C) is higher compared to other types of glass (500–600 °C). As a result, the existing mould coatings used for precision glass moulding have a shorter lifespan when subjected to the temperature required for moulding borosilicate glass [4].

One potential option is the utilization of glassy carbon, a high-temperature material that possesses a combination of features resembling both graphite and diamond. Notably, glassy carbon exhibits chemical and physical characteristics that eliminate the need for a coating [5,6]. One of the primary difficulties encountered pertains to the production of moulds utilizing glassy carbon material. The process of ultra-precision grinding, which has previously been established in the manufacturing industry for producing moulds with optical surfaces, is deemed to be a suitable method [7]. The theory of ductile-regime grinding is a frequently used approach that facilitates the achievement of defect-free surfaces through a regulated and deterministic process [8,9].

Nevertheless, the precise nature of the cutting behaviour and the subsequent formation of micro flaws during the machining of glassy carbon remains uncertain. Hence, the primary objective of this study is to comprehensively comprehend the underlying factors contributing to the occurrence of defects in the process of ultra-precision grinding of glassy carbon. This research specifically examines the cutting mechanisms involving diamond cutting edges and their impact on the surface integrity of glassy carbon.

## 2. Materials and Methods

**Figure 1:**
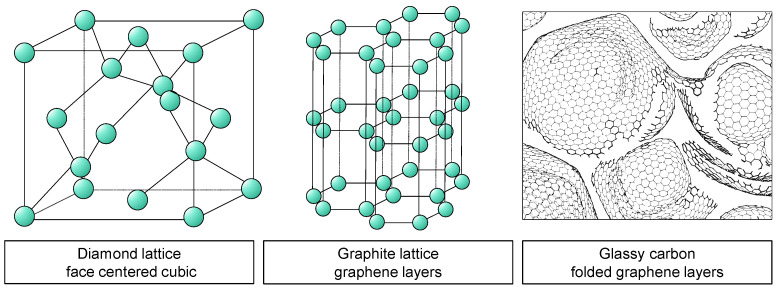
Visualization of the lattice structure of diamond, graphite, and glassy carbon.

Glassy carbon is classified as an inorganic non-metallic substance. The phrase “non-organic materials” pertains to substances that possess a structure without organic components, wherein primary heteropolar or covalent bonds are present. The interior structure comprises lattice or mesh structures, as seen in Figure 1 [10].

Carbon is found in two stable crystalline forms; namely, diamond and graphite. In the process of diamond modification, the carbon atoms arrange themselves in a face-centred cubic (FCC) lattice. The structure of diamond is one of the contributing factors to its exceptional hardness, among other characteristics [10]. Graphite is the term used to describe the most advanced and stable alteration of carbon material. Graphite exhibits a distinctive feature of having consistently organized hexagonal layer lattices, which are also referred to as graphene. Strong covalent connections exist within these levels. The interlayer bonding between the layers is characterized by a relatively low strength. The structural characteristics of the material give rise to its direction-dependent features. In contrast to the conventional layer arrangement observed in graphite, there exist diverse microstructures wherein the graphene layers exhibit a state of disorder. The materials in question are referred to as paracrystalline carbon materials [10,11].

Within these paracrystalline formations, the distribution of graphene fragments often follows a normal distribution with regard to their shape and size. The presence of flaws in the microstructure leads to the folding and interweaving of individual graphene layers in a three-dimensional manner, ultimately leading to the formation of fullerene-like structures. According to the works of Harris [12] and Jurkiewicz [13], it is evident that glassy carbon is characterized by its paracrystalline nature and closed-porous microstructure, which is mostly composed of interlaced and folded hexagonal layers. The microstructure exhibits variations contingent upon the specific manufacturing method employed, hence resulting in corresponding variations within a defined range of material attributes [14]. Currently, there is not a universally acknowledged structural framework for glassy carbon [15].

Glassy carbon has a high degree of brittleness often characterized by flat surfaces in its untreated state. According to Sharma [14], glassy carbon exhibits impermeability to a wide range of gases and liquids and demonstrates chemical inertness. Glassy carbon is a kind of carbon that is produced from the pyrolysis of polymers, and it does not form graphite [12]. During this procedure, the polymer chains undergo fragmentation due to the application of high temperatures, resulting in the creation of novel molecules involving carbon atoms exclusively. The formation of different carbon modifications or chars during pyrolysis is influenced by factors such as the polymer employed, the atmospheric conditions, and the temperature. In practical applications, the pyrolysis of glassy carbon involves the use of strongly crosslinked aromatic polymers, such as phenolic and furan resins [11].

Glassy carbon exhibits excellent corrosion resistance, impermeability to gases and liquids, and high thermal stability, withstanding temperatures of up to 550 °C in ambient air and up to 3000 °C when shielded in a protective atmosphere. The fracture toughness of the material is measured to be 20 N/mm^3/2^, while the modulus of elasticity is determined to be 35 kN/mm^2^. Glassy carbon is utilized as electrode material in laboratory technology, in medicinal technology, and in high-temperature applications in mechanical engineering due to its inherent qualities [11].

Table 1 presents a comprehensive compilation of material parameters pertaining to glassy carbon, with a comparative analysis of optical glass BK7. The machining characteristics of BK7 optical glass have been thoroughly studied and serve as a foundation for the examination of glassy carbon in this research. Despite its name, glassy carbon displays distinct material characteristics from glass. However, a comparison between the two is still valuable because of their shared brittle material behaviour.

### Indentation, Nano-Scratch, and Grinding Research Method

In the process of developing and manufacturing mechanical parts and components, mechanical parameters such as yield stress have significant importance. The mechanical properties of glass, in particular, exhibit variations based on their chemical composition and production techniques [18]. Conventional mechanical testing techniques, such as the tensile test [19], are intricate and costly, rendering them unsuitable for evaluating miniature components due to the challenges associated with specimen preparation. The methods employed in this particular case involve indentation and scratch testing. Several research studies have been undertaken utilizing the indentation method to evaluate the elastic-plastic characteristics of materials by analysing their indentation behaviour [20,21,22]. The relationship between plastic stress and indentation hardness at different strains is a valuable tool for establishing a connection and determining the yield stress and other plastic properties of metals.

Additional experimental studies utilizing modified hardness indentation tests on various optical glasses demonstrate consistent fracture propagation behaviour over a wide range of glass compositions. During the loading process, a hydrostatic compressive stress field is generated beneath the body of the pyramidal indentation [23]. The evolution of the material is characterized by both an increase in the density of the glass and the construction of a wall composed of plasticized material along the surfaces of the pyramid at a macroscopic level. Stress-relief fractures characterized by a rapid propagation velocity and perpendicular direction to the glass surface along the indentation diagonal manifest abruptly alone during the process of stress release [24]. When the glass surface experiences complete relief, the formation of fractures occurs at a relatively moderate pace. The application of pressure by the indentation body causes the glass to undergo compression, resulting in the development of radial tensile stresses. They have the responsibility for the continued dissemination of the fractures in a conchoidal form.

With the method of scratch testing, similar mechanisms of the above-mentioned also occur when a glass surface is subjected to stress during scratching. The scratch test method should be employed to examine the plastic characteristics of materials [25]. Scratching is a well-known empirical method for determining the hardness according to the Mohs scale [26,27]. Because it has a negative effect on the service life of glasses [28,29,30], if the scratch normal force does not exceed a certain level, a crack-free, plastically deformed scratch trace can be produced. Due to the stresses induced in the vicinity of the scratch by the compaction zone, such plastic scratches are highly unstable and tend to form cracks. The cracks spread into the existing plastic part of the scratch and form conchoidal side cracks. 

The investigation of brittle behaviour is commonly conducted using various materials by indentation and nano-scratch techniques; therefore, Bifano’s model is frequently utilized as a reference for determining the critical depth of cut in ductile-regime machining of brittle materials [8]. Nevertheless, the model solely relies on material characteristics and does not include factors like machining conditions, tool shape, and size [31]. Adjusting the process settings to decrease the chip thickness leads to improved surface quality, as it approaches or decreases below the critical chip thickness for ductile grinding [32,33,34,35]. The alteration of the grain size of the grinding tool also affects the resulting chip thickness. Smaller diamond grains lead to improved surface roughness and might potentially induce complete plastic material behaviour [35]. However, further examinations utilizing nano-scratching techniques in gallium and silicon demonstrate that the essential cutting depths varied depending on whether conical or Berkovich tips are employed [36]. Modifying the lubrication quantity can also result in corresponding effects on the critical chip thickness [37], unrelated to the material properties. The anisotropy of the material significantly affects the damage evolution and material removal behaviours during the machining process [38]. 

**Figure 2:**
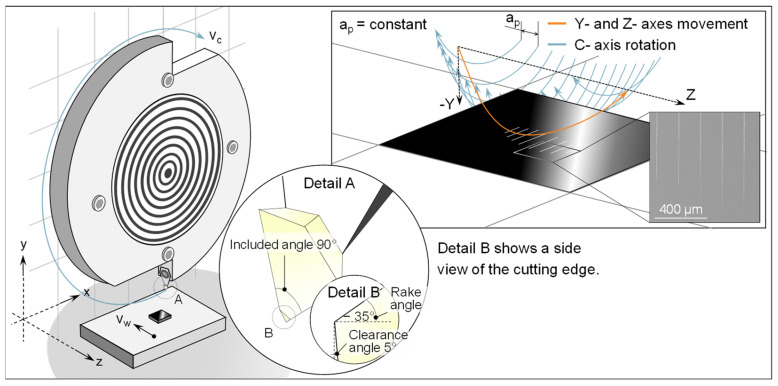
Visualization of the scratch test setup and kinematics.

To examine the aforementioned processes in glassy carbon, a series of traditional Vickers hardness tests and nano-scratch examinations were conducted on prepared polished samples. The schematic technique represented in Figure 2 enables the investigation of whether glassy carbon has comparable brittle material behaviour. The scratch examinations were conducted at several cutting speeds to yield results for the subsequent grinding research. Furthermore, a scratch trace was subjected to calotte grinding in order to observe the presence of the separation phenome in the subsurface.

The motion of the cutting edge in the scratch tests may be described as a helical route. In this setup, the feed movement with the workpiece velocity v_w_ is achieved by utilizing the z-axis (movement direction: −z), on which the glassy carbon sample is securely affixed to a sample holder. The helix undergoes a circular motion due to the continuous rotation of the spindle. The helix’s central axis is curved towards the sample by a concurrent semicircular motion in the negative y direction. If there is enough contact with the sample surface, the cumulative movement of all axes will create scratches on the surface of the sample. These scratches will vary in length and depth. Please refer to Figure 2 in the upper right corner for a visual representation. As a result of the continuous feed along the *z*-axis, all scratches have an equal path distance a_p_. The experiments were conducted using a typical UP-grinding machine; namely, the Nanotech 350FG model (Moore Nanotechnology Systems, LLC, Swanzey, NH, USA). In order to obtain the necessary cutting speeds v_c_, a customized tool holder was devised and affixed to the machine’s C-axis spindle. Performing scratch testing on the same grinding machine can enhance the capacity to replicate the grinding process behaviour, as it also replicates factors such as machine vibrations. The uniform machine environment should further enhance the transferability to other experiments in this publication. A sharply cut diamond from a turning tool was used as the abrasive grain. A diamond with an included angle of 90° and a rake angle of −35° was used to simulate the geometry of a diamond grain.

**Figure 3:**
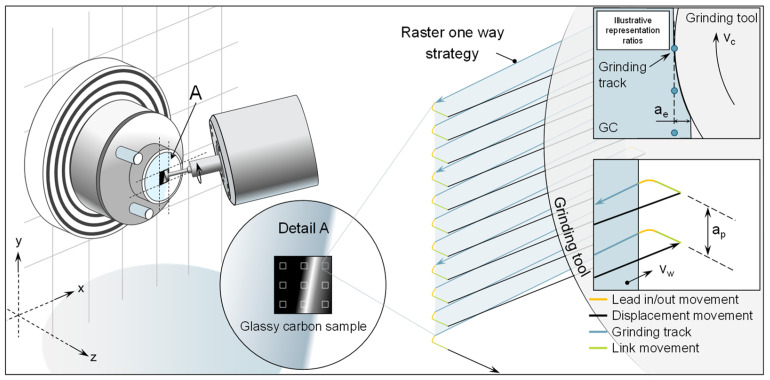
Visualization of grinding test setup and strategy.

A restriction in the investigation of material removal mechanisms by means of indentation or scratch tests is that only the singular intervention of a grain can be investigated. In the grinding process, however, there are interactions and overlaps of engagement paths of differently shaped diamond grains, which can thus constantly change the removal mechanisms. Consequently, a series of comprehensive grinding technology experiments was conducted. The experiments were conducted using a 5-axis ultra-precision grinding machine. The machine is situated inside a regulated setting characterized by a consistent temperature (T = 21 ± 0.1 °C) and relative humidity (40 ± 5%). The schematic representation of the experimental system is illustrated in Figure 3. Liquid isoparaffin is employed as a cooling agent in the context of minimal amount lubrication. The use of isoparaffin has become widely accepted as the prevailing method in ultra-precision machining [39].

The glassy carbon Sigradur G utilized in this study was procured from HTW Hochtemperatur Werkstoffe GmbH (Thierhaupten, Germany) [16]. The specimens exhibit a square shape, measuring 10 mm × 10 mm in length and width, with a thickness of 3 mm. In order to provide equitable testing conditions and reduce any potential surface imperfections, the specimens undergo a preliminary grinding and polishing process. The resultant initial surface has a consistently polished appearance, characterized by roughness values Sq that are less than 2 nm.

Prior to each usage, the grinding wheels undergo dressing to achieve a tip radius of 400 µm. In order to avoid the grinding tools from becoming unclamped from the grinding spindle during the dressing process, they are dressed on a dedicated dressing spindle that is mounted within the machine. This offers the benefit of utilizing high-precision machine axes to achieve precise dressing. The dressing procedure consists of two stages. Prior to the application of the dressing, any irregularities are eliminated and the proper curvature is created using a truing procedure. Subsequently, in the process of dressing, a portion of the bond is eliminated to reveal the sharp cutting edges of the abrasive grains. These two steps are executed in accordance with the instructions provided by the manufacturer. Table 2 displays the truing and dressing parameters.

The grinding trials are conducted using a flat surface grinding technique. In this scenario, the grinding motion is performed in a raster pattern. In this experiment, it is important to highlight that the relative motion between the workpiece and the grinding tool is produced by the movement of the workpiece itself, with a velocity denoted as v_w_, along the x and y machine axes. The movement of the grinding tool (z machine axis) only determines the cutting depth, represented by a_e_. The raster movement in these trials begins in the upper right corner of the machining surface. The cutting tool moves towards the surface of the sample in a linear link motion. A second lead-in motion decreases the velocity of the link motion and guarantees a seamless and accurate transition to the grinding motion and additionally minimizes the tool load in transit. The grinding movement, which involves the workpiece moving in the positive x direction, follows a straight-line trajectory. After this, there is a further movement known as a lead-out and link. The grinding tool is moved to the right-hand side of the sample using a linear displacement motion. The workpiece undergoes motion in the negative x-direction and positive y-direction. Using this kinematic configuration, the vertical displacement of the glassy carbon sample results in the grinding path distance, indicated as a_p_. This process is iterated until a segment of the sample is fully ground. The combination of the grinding wheel’s direction and rotating cutting velocity v_c_ along with the movement of the workpiece, leads to cross-axis kinematics with a down-cutting direction. The generation of the NC code is facilitated by the utilization of NanoCam4 (Version 4.2309.9) and is visualized in Figure 3.

Based on the application as mould material for precision glass moulding, the grinding parameters were chosen to investigate the influence of tool properties and process control variables. The parameters are based on a cross-axis rotational process which is commonly used in mould manufacturing for optics. Further, the chosen parameters are additionally based on investigations from the literature [40,41,42]. Table 3 gives an overview of the investigated parameters, which includes tools with different diamond grain sizes D3 and D9 and varying cutting speed and feed rates. Other parameters are kept constant, like grain concentration, cutting depth, and path distance.

## 3. Results and Discussion

### 3.1. Indentation and Scratch Investigation

**Figure 4:**
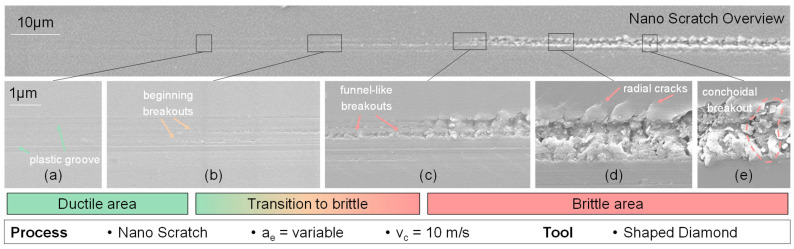
SEM pictures of nano-scratch behaviour from ductile to brittle (**a**–**e**).

To investigate the primary attributes of scratching on glassy carbon, a sequence of individual scratch experiments was performed and afterwards analysed utilizing scanning electron and atomic force microscopy. The behaviour of each scratch has a notable correlation with the extent of cutting depth. At reduced cutting depths, the scratch exhibits mostly ductile behaviour, as indicated by the substantial plastic deformation revealed in Figure 4a and beginning small minor additional scratches in the contact zone (Figure 4b). The surface of the scratch exhibits radial cracks that are inclined in a forward direction, resulting in a funnel-shaped formation (Figure 4c). The wider opening of this funnel formation is oriented towards the direction of the scratch mark’s progression.

As the magnitude of the depth of cut is increased, the occurrence of radial fractures propagates throughout the contact area at the outer margins of the ductile region (Figure 4c). An additional increase in the depth of the cut results in the generation of large beginning breakouts, accompanied by the presence of partially fragmented glassy carbon chips that stay inside the scratched area (Figure 4d). The radial fractures exhibit partial widening and form a beginning chip which is not removed. As a result, an increasing number of fragmented chips become apparent, and the expanded radial fractures give rise to bigger breakouts that extend to the farthest points of the radial cracks. After this stage, observable lateral fractures become evident, leading to the formation of clod-like breakouts that replace the funnel-shaped breakouts (Figure 4e). The distinguishing factor between funnel-shaped and clod-like breakouts lies in the character of their contact zones. The funnel-shaped breakouts are confined to the scratch region, but the clod-like breakouts exhibit larger dimensions compared to the chipping zone.

**Figure 5:**
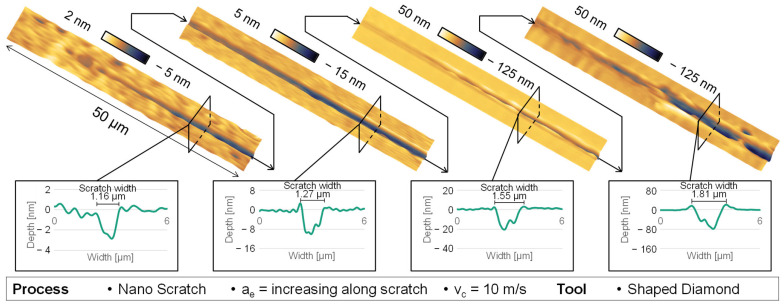
AFM and cross-section measurement of nano-scratch behaviour.

Moreover, by the analysis of the two-dimensional cross-section profiles of the scratch along the track, it is possible to analyse the plastic behaviour at different depths of cut as seen in Figure 5. In general, the resulting scratch depth is overproportioned to the real cutting depth of the tool, which indicates brittle behaviour at deeper scratch depths. In the context of purely plastic deformation at the beginning of the scratch, there are no disturbances or disruptions, and the absence of chip formation indicates that the plastic deformation is only characterized by compressive material alteration. In the scenario of emerging radial fractures, which starts on the second left AFM image in Figure 5, there is no observable material displacement occurring at the outer boundaries of the scratch track, which indicates that the crack propagation is probably further outside than the plastic deformation area. The elevation of quantifiable material along the scratch is observed exclusively after the formation of radial fractures at the beginning scratch width of 1.27 µm and scratch depth of 9–10 nm, which is already in the range of brittle behaviour. 

**Figure 6:**
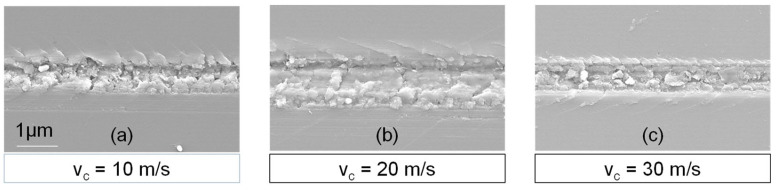
Visualization of radial crack inclination in varying cutting speeds. v_c_ = 10 m/s (**a**). v_c_ = 20 m/s (**b**). v_c_ = 30 m/s (**c**)

An additional analysis of the scratch marks produced at varying cutting speeds reveals that the material’s behaviour remains consistent (Figure 6). However, it is seen that the formation of radial cracks is more inclined in the cutting direction, leading to clod-like breakouts that cover a narrower angle. This indicates that the crack propagation and material removal can be controlled with the cutting speed and it does show deterministic behaviour similar to the general ductile and brittle behaviour with increasing cutting depth.

**Figure 7:**
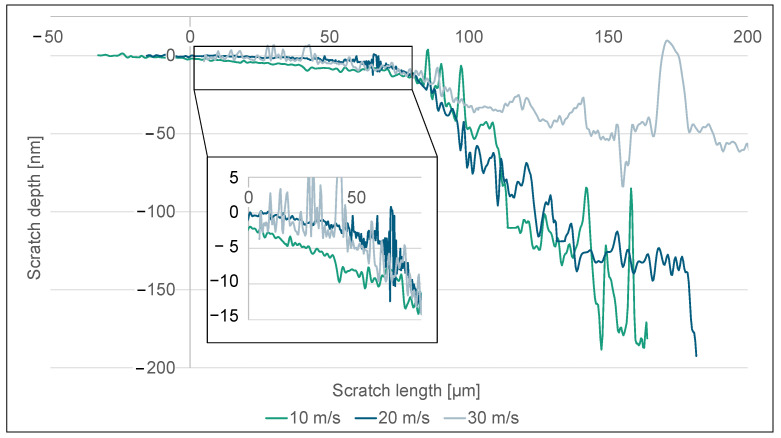
Scratch depth along the scratch length with varying cutting speeds.

Further examination of the depth of scratches along the path of the scratch verifies the previously discussed phenomenon, as seen in Figure 7. The observed scratch depths were adjusted in a manner that aligns with the initiation of the brittle zone when there is a notable rise in scratch depth. It is worth noting that during the transition to the brittle-hard zone, there is a substantial rise in fracture depth, which can be explained by the breakouts and crack propagation exceeding the cutting depth of the diamond. At a cutting speed of 30 m/s, there is evidence of a notable decrease in fracture depth inside the brittle region compared to lower cutting speeds. This indicates a reduction in the propagation of cracks at greater depths, which in turn facilitates the early emergence of lateral cracks. Furthermore, there is a noticeable variation in depth within the ductile area. When the cutting speed is set to 10 m/s, it is noticed that the plastic zone exhibits increased length and depth. At lower cutting speeds, the plastic deformation strain is increased, leading to the occurrence of incipient separation fractures at a later stage. The region exhibiting exclusively ductile behaviour becomes apparent when the cutting speed is set at 10 m/s, resulting in a scratch depth of 5 nm. Similarly, with cutting velocities of 20 and 30 m/s, the highest ductile scratch depth seen is limited to 3 nm.

**Figure 8:**
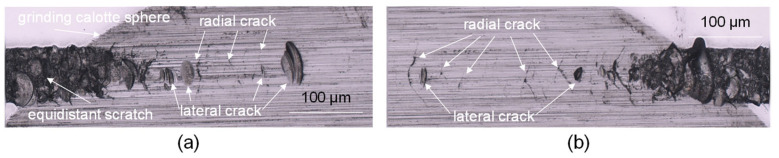
Subsurface investigation of nano-scratch revealed by calotte grinding. Subsurface damage at beginning of scratch (**a**) and end of scratch (**b**).

To further examine the fracture behaviour in the subsurface zone, an additional scratch track was created with equidistant cutting depth, resulting in brittle breakouts. Subsequently, the subsurface area was removed using sphere calotte grinding to analyse the radial and lateral crack behaviour in different depths below the surface as shown in Figure 8. The presence of radial fractures is predominantly seen to be arranged either perpendicular or at an oblique angle relative to the cutting direction. In certain instances, the radial fractures exhibit a flared angle. The clod and shell-like breakouts exhibit shallower depths compared to the radial fractures and are typically situated posterior to a radial crack along the direction of the section. The conchoidal fractures, also known as lateral fractures, occur when a crack originates at a minor angle to the surface and extends until the chip breaks away entirely, causing the crack to propagate back towards the surface.

**Figure 9:**
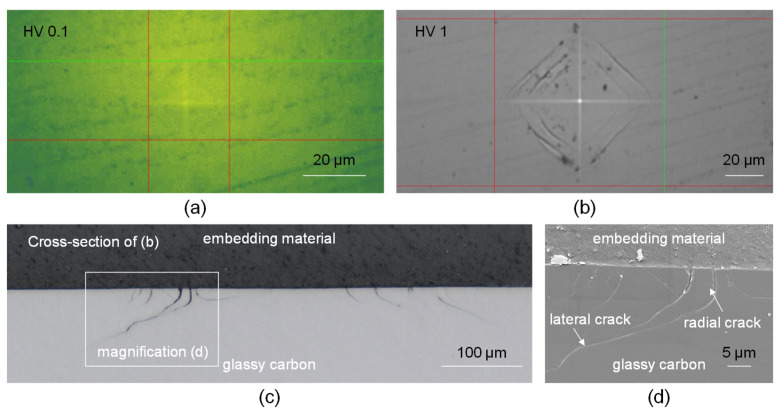
Vickers indentation crack behaviour and cross-section view. HV0.1 (**a**). HV1 (**b**). Cross-section view of HV1 indentation (**c**,**d**).

Additional indenter examinations including varying magnitudes of the Vickers pyramid forces revealed an absence of observable fractures, with only an evident indication of plastic behaviour (Figure 9a). At lower levels of applied indentation forces, the absence of any apparent indentation can be identified as the elastic response shown by the material. In addition, it can be observed that there were no radial cracks observed around the edge of the pyramid, indicating a characteristic of low residual stress. Similar to the subsurface crack propagation of a single scratch, the same pattern of activity is also observable in the indentation experiments at higher indentation forces. The clod-like breakouts that may be observed are apparent along the sides of the Vickers pyramid when viewed from the top (Figure 9b). Upon seeing the subsurface through a cross-sectional view, it becomes apparent that the edge zone has radial fractures that diverge from the sides of the pyramid and extend laterally deeper inside the material (Figure 9c,d). The observed phenomenon aligns with the conchoidal breakouts identified during the subsurface scratch test, as they progressively extend horizontally into the subsurface zone until reaching the removal of the chip. Likewise, the presence of plastic deformation in the central region is apparent, as shown by the observable Vickers pyramid. Subsequent examinations including varying magnitudes of the Vickers pyramid forces revealed an absence of observable fractures, with only an evident indication of plastic behaviour (Figure 9a). At lower levels of applied indentation forces, the absence of any apparent indentation can be identified as the elastic response shown by the material. In addition, it can be observed that there were no radial cracks observed around the edge of the pyramid, indicating a characteristic of low residual stress.

### 3.2. Grinding Investigation

**Figure 10:**
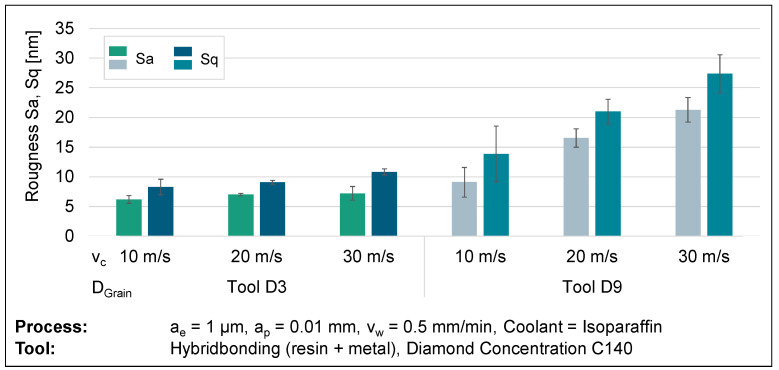
Surface roughness results from varying cutting speeds and diamond grain sizes.

A study was conducted to investigate the grinding of glassy carbon, as scratch tests do not consider the interaction between several diamond grains in contact. The inquiry focused on analysing the material’s behaviour through the manipulation of process parameters, including the cutting speed, feed rate, and diamond grain size of the tool. In order to establish a precise and objective comparison, an individual variable was deliberately altered and then evaluated for comparison. Statistical reliability was established by conducting a minimum of three tests for each parameter set. The test specimens were subsequently analysed for surface topography using a confocal microscope, scanning electron microscope, and atomic force microscope. An analysis of surface roughness was conducted to determine a relationship between process parameters and surface structure. Topographical imagery can aid in the verification of meteorological results. An analysis of roughness metrics Sa and Sq at cutting speeds of 10, 20, and 30 m/s demonstrates a noticeable pattern of heightened roughness with higher cutting speeds. The visual representation of this discovery can be seen in Figure 10. Further grinding wheels with an average diamond grain diameter of 3 µm (D3) have lower surface roughness compared to grinding wheels with a relatively larger average diamond grain size of 9 µm (D9). This supports the common belief that an increase in grain size leads to thicker chips, resulting in larger breakouts and more brittle behaviour. Figure 10 demonstrates the impact of increasing roughness by a factor of more than two, from D3 to D9, on grain size at a velocity of 30 m/s.

**Figure 11:**
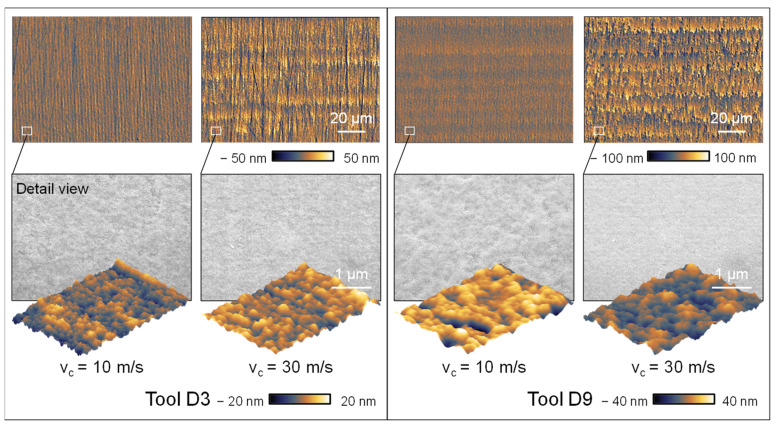
Topographic images and detail view of varying cutting speeds and diamond grain sizes.

This discovery contradicts the widely known assumption in the literature that greater cutting speeds lead to smaller chip removal and therefore result in improved roughness values [43,44,45,46,47]. Upon closer analysis of the surface topography depicted in Figure 11, it becomes apparent that increasing the cutting speed does indeed lead to an increase in surface roughness. Nevertheless, as the cutting speed is increased, the surface shows disruptions and a higher frequency of imperfections or slight chipping. At higher cutting speeds, the individual paths become more noticeable due to an increased accumulation of material. Upon closer examination, it is observed that the pile-up structures at higher cutting speeds exceed the anticipated kinematic roughness. This phenomenon cannot be accounted for by the plastic deformation of the material. Hence, the observable accumulation formations are formed due to the tool wear of the cutting tool, which accounts for the rise in surface unevenness at elevated cutting velocities.

**Figure 12:**
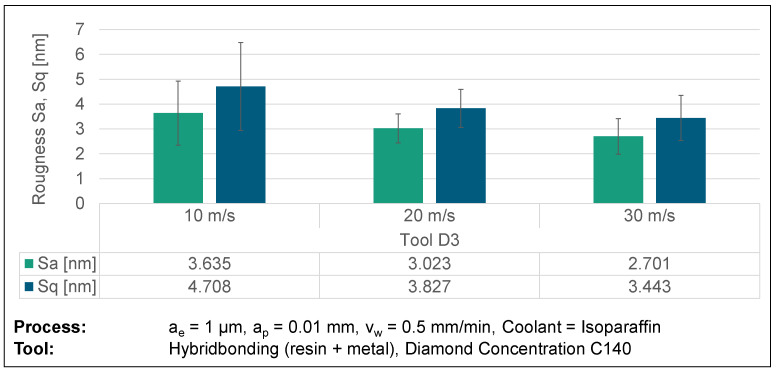
AFM surface roughness results from varying cutting speeds.

Upon analysing the grinding topography observed in the SEM and AFM images, higher cutting speeds result in the formation of thinner and more distinct grinding marks caused by the rotation of the grinding wheel. This observation is further confirmed by the thorough analysis of the surface topographies. Increasing the cutting speed creates a surface topography characterized by less chipping and a more homogeneous visual aspect. The validity of this assertion is confirmed by the utilization of atomic force microscopy (AFM) to measure the roughness of the surface. This is demonstrated in Figure 12, which showcases the findings acquired through the utilization of the D3 grinding wheel as a specific example. The AFM images show a mostly consistent surface; however, at a velocity of v_c_ = 10 m/s, the surface displays the presence of overlapping irregular globular breakouts. Enhancing the cutting speed leads to a significant diminishment in the dimensions of globular breakouts, together with a decline in their depth, as confirmed by the AFM roughness measurements. Hence, it can be determined that a higher cutting speed leads to an improvement in the surface roughness. The presence of tool wear, caused by the elevated cutting speeds, shows a more pronounced influence on the resultant roughness than the cutting speed alone.

The results of scratch tests indicate that increasing the cutting speed causes the funnel-like and conchoidal breakouts to occur in a narrower separation fracture. This finding further supports the idea that higher cutting speeds result in lesser surface roughness. Nevertheless, there are no discernible directional structures, such as funnel shapes or other patterns, that may be observed on the ground surface. The absence of uniform contact conditions among the abrasive grains leads to unregulated breakouts, indicating the occurrence of fracture propagation in both perpendicular and parallel cutting directions. However, breakouts at higher cutting speeds exhibit a reduced form factor, which improves the surface roughness. The fractures, which are less than 1 µm in size, indicate that the material removal occurs in the contact zone between the diamond grain (3 µm and 9 µm) and the glassy carbon. This implies a material removal behaviour within the transition zone from ductile to brittle, with no apparent radial surface cracks present (Figure 4b,c).

**Figure 13:**
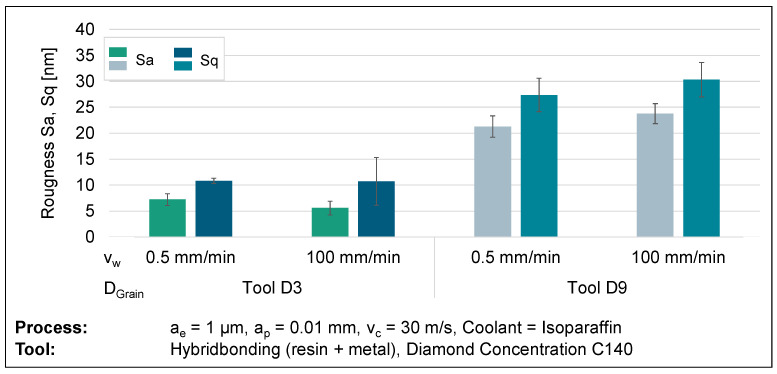
Surface roughness results from varying feed rates and diamond grain sizes.

Further examination of the impact of the feed rate reveals contrasting patterns in relation to the diamond grit sizes used while maintaining a constant cutting speed of v_c_ = 30 m/s. An improvement in roughness is observed with an increase in feed rate for smaller diamond grit sizes (D3), whereas a rise in surface roughness is noted for larger diamond grit sizes (D9). This phenomenon is seen in the roughness values depicted in Figure 13.

**Figure 14:**
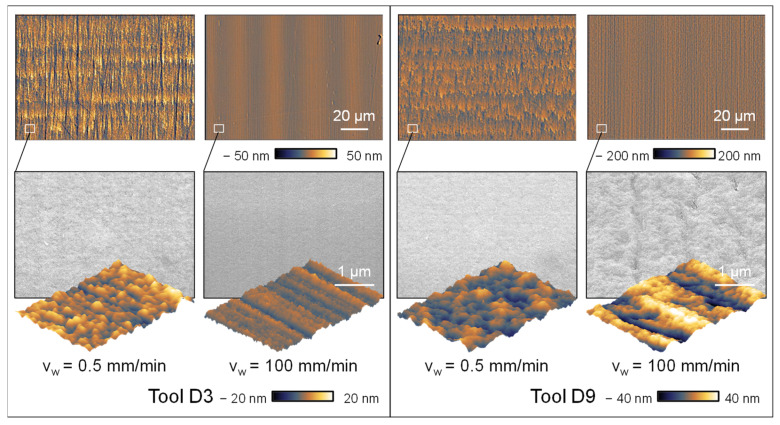
Topographic images and detail views of feed rates and diamond grain sizes.

The examination of the topography images validates the observed roughness values, as it is seen that both the macroscopic roughness and the microstructure exhibit consistency. The accepted hypothesis regarding the D9 grinding wheel suggests that there is a positive correlation between the feed rate and the chip thickness, thus resulting in higher roughness when increasing the feed rate. The D3 grinding wheel exhibits an inverse behaviour. The microstructure (D3) seen at a feed rate of v_w_ = 100 mm/min has a high degree of homogeneity on its surface, characterized by the presence of isolated break-out formations of minimal size, as depicted in Figure 14. Conversely, an observable phenomenon of elongated fractures in the grinding direction becomes apparent when the feed rate increases on the D9 grinding wheel. Furthermore, the surface on both grain sizes exhibits an oscillating structure, likely attributed to the machine tool’s control mechanism. Nevertheless, it has no substantial effect on the roughness measurements.

The improvement of surface topography achieved through the utilization of a D3 grinding wheel may be defined by the impact of the bonding agent on the grinding tool. The grinding wheel bond exhibits a more uniform distribution when the concentration of diamonds remains constant, but the average diamond grain diameter decreases. This phenomenon facilitates increased deformation of the grinding wheel, resulting in elevated surface pressure on the material being ground. This phenomenon promotes the plastic deformation of glassy carbon and prevents or limits the spread of cracks. In the context of the D9 grinding wheel, apart from the unique behaviour resulting from the bond, the increased chip thickness can also induce greater brittleness. Higher feed rates evidently result in more visible grinding marks on the direction of the rotating grinding wheel. The grinding marks also indicate more ductile grinding behaviour in the case of a D3 tool as seen in the nano-scratch investigations (Figure 4b). Additionally, the AFM image of the close-up shows a similar pattern at v_w_ = 100 mm/min as investigated in the nano-scratch test and indicates ductile behaviour with plastic flow, because of the visible grinding marks. In the case of D9 tools, the overall removal mechanism shows brittle behaviour, most likely due to the more dominant effect of increased chip thickness and wider grinding marks based on the higher diamond grain size. 

Additionally, the occurrence of pile-ups in the feed rate direction are not evident which indicates less tool wear due to the overall short use of the grinding wheels with feed rates v_w_ = 100 mm/min and which supports the aforementioned influence of the tool wear at lower feed rates.

**Figure 15:**
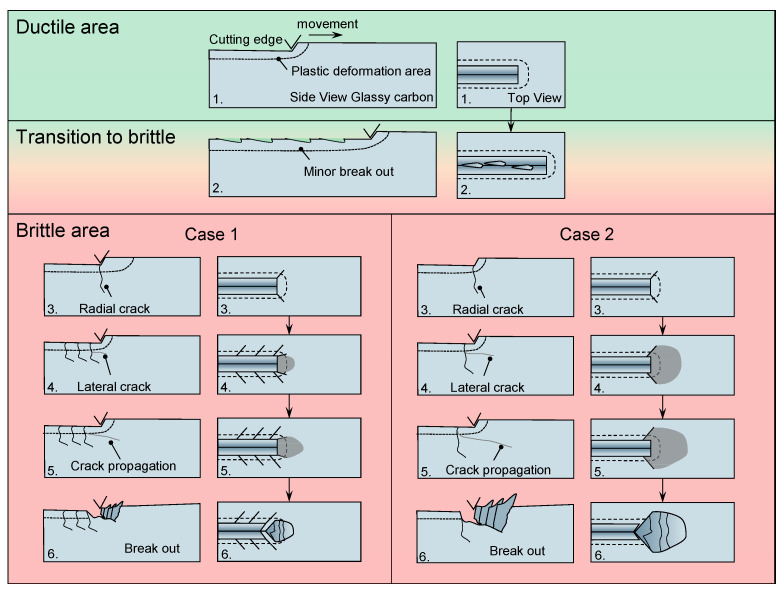
Crack propagation and breakout behaviour of glassy carbon. Increasing cutting depth from 1–6. Only plastic deformation (ductile) 1. Beginning break outs (transition) 2. Crack behaviour (brittle) 3–6.

The findings indicate that the material response of glassy carbon during ultra-precision grinding is characterized by a high degree of complexity, with many factors typically interacting simultaneously. The scratch tests facilitated the identification of fundamental material behaviour, hence providing some insight into the grinding process. To enhance comprehension, Figure 15 was utilized to reconstruct the crack propagation seen during scratch testing, based on the obtained data. 

Once the elastic behaviour of glassy carbon is surpassed, the material undergoes plastic deformation and compression. Once the plastic deformation capacity is surpassed, the initial fractures to emerge are minor funnel-like breakouts that probably have their initiation due to a radial crack which transforms into a lateral crack. The brittle behaviour shows two cases which differ in the area of effect. Both cases start with radial cracks that are orthogonal to the cutting direction and exceed the plastic deformations area. As the cutting edge progresses, a lateral fracture emerges, originating at an elevated position due to the surpassing of shear forces over the tensile strain in this region. The convergence of the lateral crack and radial fractures culminates in the formation of a conchoidal breakout. In case 1, the breakout is not formed by the outmost radial cracks compared to case 2. In the general case of brittle-hard behaviour, the radial cracks exhibit crack propagation that extends beyond the depth of cut. In the context of the ductile zone, it is unlikely that fracture propagation extends beyond the plastic region. The confirmation of this phenomenon may be obtained by the examination of the grinding process, where mostly globular chipping is observed. This chipping does not exhibit any discernible fracture development that follows a certain path. Furthermore, the presence of a consistently identifiable structure provides evidence for a machining process that operates within the ductile range.

## 4. Conclusions

In conclusion, this study could demonstrate the general behaviour of glassy carbon under abrasive machining conditions. Specifically, the fracture behaviour may be systematically studied and comprehensively explained. The findings obtained from the scratch experiments may be used for the grinding process, therefore providing a basis for understanding potential material behaviour. The nano-scratching process initially exhibits ductile plastic deformation, which is then followed by the occurrence of funnel-shaped breakouts in the contact zone as the cutting depth increases. Additionally, increased cutting depth results in mostly brittle material behaviour characterized by the initiation of radial fractures that then propagate into lateral cracks, resulting in the formation of conchoidal breakouts. The impact of process parameters and other tool-related parameters induced by grinding has distinct effects on the final surface roughness and topography. Through the extraction of microtopography, it can be demonstrated that the overall predicted grinding behaviour aligns with the general assumptions presented in the literature. Increasing the cutting speed while using cross-axis kinematic grinding leads to improved surface roughness. Conversely, increasing the diamond grain sizes and feed rates have the opposite effect, causing an increase in surface roughness. The primary factor influencing the roughness is the size of the diamond grains, whereas the impact of cutting speed and feed rate is minor. In addition, roughness values of less than 5 nm can be achieved with homogeneous surface topography, which makes it possible to use glassy carbon as a mould material. Nevertheless, the tool wear has a substantial influence on the roughness of the surface when using hybrid bonding (metal-resin) tools at reduced feed rates. The decrease in tool diameter can result in pile-up structures that exceed the theoretical kinematic roughness. This phenomenon bears great significance in rotational grinding processes, which are frequently employed in the fabrication of optical lenses. Typically, these processes include operating at low rotating speeds while cutting the central region of the optical mould surface. Hence, the increased surface roughness might restrict the use of glassy carbon moulds. The potential for ultra-precision ground glassy carbon moulds is significant when using other three-axis ground surfaces with constant greater feed rates, such as moulds for inclined cover glasses.

In addition to tool wear, the bonding agent itself has a beneficial effect on the surface roughness when higher feed rates are used in combination with small diamond grit sizes. This supports the theory that the resulting compressive stress leads to more ductile behaviour, as it reduces the ability of cracks to propagate and initiate.

However, the researchers also suggest that it is crucial to undertake further investigations, such as subsurface zone analyses and modifications of additional process parameters, in order to fully comprehend the material behaviour in ultra-precision grinding. Conducting research on process parameters involving increased cutting speed and feed rates, while simultaneously monitoring the effects of tool wear, might be beneficial in utilizing glassy carbon as a material for moulds.

These additional studies can provide new insights into the behaviour of separation fractures and the process of ductile machining. Furthermore, the impact on precision glass moulding with glassy carbon based on the resulting surface roughness induced by grinding has not yet been investigated.

## Figures and Tables

**Table 1:** Material properties of glassy carbon and BK7 [16,17].

Material Property	Glassy Carbon Sigradur G	Optical Glass BK 7
Characteristic temperature	>3000 °C (decomposition)	557 °C (transition)
Density	1.42 g/cm^3^	2.51 g/cm^3^
Hardness	2.26 GPa	5.80 GPa
Young’s modulus	35 GPa	82 GPa
Thermal conductivity	6.30 W/(mK)	1.11 W/(mK)
Coefficient of thermal expansion (20–200 °C)	2.6 × 10^−6^ /K	8.3 × 10^−6^ /K

**Table 2:** Truing and dressing parameters.

Parameter	Truing (Green Corundum)		Dressing (White Aluminium Oxide)	
Relative wheel speed	100 m/min		50 m/min	
Cutting direction	Down Cut		Down Cut	
Cutting depth (a_e_)	10 µm		5 µm	
Feed rate (v_w_)	20 mm/min		10 mm/min	
Grinding wheel	#1200 (D9)	#3000 (D3)	#1200 (D9)	#3000 (D3)
Truing/Dressing wheel	#600	#600	#320	#600

**Table 3:** Grinding process parameters.

	Parameter	Unit	Value
Kinematic	Cross-Axis	-	-
Tool properties	Diamond grain size/ Mesh size	[µm]	D3/#3000, D9/#1200
	Concentration	-	C140
	Bonding type	Hybrid (metal-resin)	-
Process parameters	Cutting speed	[m/s]	10, 20, 30
	Feed rate	[mm/min]	0.5, 100
	Cutting depth	[µm]	1
	Path distance	[mm]	0.01

## Data Availability

Data is contained within the article.
